# Bioprospecting of Palmyra Palm (*Borassus flabellifer*) Nectar: Unveiling the Probiotic and Therapeutic Potential of the Traditional Rural Drink

**DOI:** 10.3389/fmicb.2021.683996

**Published:** 2021-06-28

**Authors:** Nagamani Pammi, Kiran Kumar Bhukya, Ravi Kumar Lunavath, Bhima Bhukya

**Affiliations:** Centre for Microbial and Fermentation Technology, Department of Microbiology, University College of Science, Osmania University, Hyderabad, India

**Keywords:** probiotics, lactic acid bacteria, toddy palm nectar, volatile fatty acids, amino acids

## Abstract

The present study investigates the therapeutic and probiotic attributes of traditional Toddy Palm Nectar (TPN). Glucose was found to be the highest with 4.37 mg/ml and arabinose was the least with 2.85 mg/ml. The average ethanol concentration of fresh TPN was found to be 0.3 mg/ml. The nutritional profile of TPN revealed 18 volatile fatty acids, the major one being hexadecenoic acid (M/Z 74). Amino acid profiling showed 26 amino acids, with OH-lysine-2 the highest (12.86%). About 120 morphologically distinct lactic acid bacteria (LAB) were isolated from 26 TPN samples, based on differential growth and *in vitro* probiotic characteristics. After 16S rRNA sequencing, four indigenous LAB strains were identified as *Lactobacillus plantarum* group OUBN1, *Enterococcus faecium* OUBN3, *Pediococcus acidilactici* OUBN4, and *Pediococcus pentosaceous* OUBN5 and their sequences were deposited to NCBI. Microbiological safety evaluation studies showed the absence of hemolytic, gelatinolytic and proteolytic activity. The bacterial isolate OUBN3 showed a maximum survival rate of 6.91 ± 0.04 log cfu/ml at acidic pH 2.5 and isolate OUBN5 showed 6.94 ± 0.02 log cfu/ml at pH 3.0. Similarly, the isolate OUBN5 showed 7.92 ± 0.03 log cfu/ml to 0.3% ox-bile after 4 h and 8.94 ± 0.03 log cfu/ml to simulated gastric juice after 3 h of treatments. OUBN1 expressed the highest autoaggregation (81.76 ± 1.25%), cell surface hydrophobicity (79.71 ± 3.42%), and displayed the maximum coaggregation with *E. coli* MTCC452 (76.96%), *K. pneumoniae* MTCC109 (75.62%), and *S. aureus* MTCC902 (70.69%). All strains showed significant antibiotic and antimicrobial activity. Isolate OUBN1 displayed hydroxyl radical scavenging activity (68.71 ± 1.0%) with an IC_50_ value of 75.62 μg/ml and the highest anti-cancer activity (percentage inhibition of 88.55) against HT-29 cells. Based on the characteristics observed, *L. plantarum* group OUBN1 and *P. pentosaceous* OUBN5 were found to be potential isolates to employ as probiotic microbiota in food and forage preparations. These findings reinforce the fact that LAB isolated from TPN could be exploited as an alternative means toward potential therapeutic applications.

## Graphical Abstract

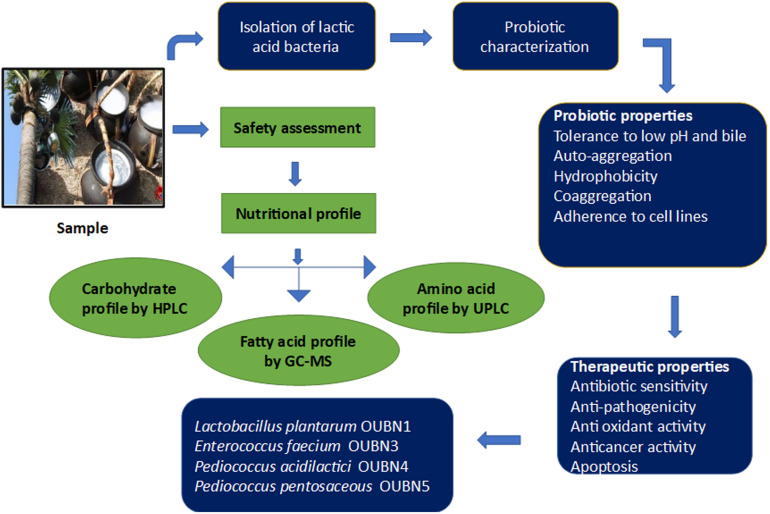

## Introduction

Fermented plant-based beverages have been persistent in human societies for ages (Lamba et al., 2019). Among them, toddy palm nectar (TPN) is one of the renowned naturally fermented seasonal traditional alcoholic beverages consumed in various regions of rural India. Due to growing lactose intolerance and allergy to milk and milk-based products, consumers are intensely demanding functional probiotic products from natural sources such as fruits, vegetables, and cereals (Sornplang and Piyadeatsoontorn, 2016).

Therefore, more attention toward fermented products of plant origin is a need of the hour (Coutiño et al., 2020). It is noteworthy that TPN represents a traditional rural drink of the Indian society for ages. Owing to the vital role it has occupied in traditional culture, it is necessary to understand the microbiological and biochemical nature of TPN, as not many studies have been conducted on probiotics and nutritional aspects of it.

TPN is a naturally fermented sap from young and matured inflorescences of *Borassus flabellifer* Linn. (Palmyra Palm), belongs to the family *Arecaceae*, and is commonly referred to as “toddy” (Zeid and FarajAlla, 2019). As a traditional energizing drink with significant health-promoting effects, TPN is enjoyed by people in parts of South America, Africa, and Asia (Reshma et al., 2017). Local names of the product include kallu in southern India, emu, and ogogoro in Nigeria, nsafufuo in Ghana, and tuba in Mexico. Fresh TPN is a colorless sweet-flavored drink with a pH between 6.0 and 7.0, which contains various carbohydrates and proteins. Generally, it contains reducing and non-reducing sugars, ethanol, and various nutrients including volatile fatty acids (VFAs), amino acids, and flavonoids (Lasekan and Abbas, 2010). It is also a good source of ascorbic acid, nicotinic acid, vitamin A, riboflavin, several minerals, and salts (Morton, 1988; Chinnamma et al., 2019). As a folk medicine, TPN obtained from the flower stalks of palm trees can be used as a tonic, stimulant, laxative, diuretic, anti-phlegmatic, and amebicide (Lim, 2012; Mariselvam et al., 2020).

Volatile fatty acids have several applications in the food, healthcare, and pharmaceutical industries and their derivatives are used as anticonvulsants in neurodegenerative diseases as neuroprotective agents (Lei et al., 2016). VFAs help to regulate insulin secretion and indirectly affect cholesterol synthesis. Plant-derived amino acids are important dietary bioactive components for human and animal nutrition. Essential amino acids have been recognized as nutrient regulators in muscle protein synthesis and tissue regeneration. Therefore, estimation of these nutritional attributes is essential for the development of plant-based foods (Tayade et al., 2017).

Fresh TPN collected under hygienic conditions during the early hours of a day is known to contain various probiotic strains of yeast and bacteria (Somashekaraiah et al., 2019). It is reported that TPN could be a potential source of probiotic LAB like *Lactobacillus, Lactococcus, Leuconostoc, Enterococcus, Pediococcus, and Streptococcus*, etc. (Amoa-Awua et al., 2007; Somashekaraiah et al., 2019). Bacteria related to *Lactobacillus*, *Pediococcus*, and *Bifidobacterium* are predominant groups used in many dietary supplements and functional foods (Pandey et al., 2015). Ideal probiotics must exhibit *in vitro* characteristics, such as tolerance to low pH, bile, gastric juice, antibiotics and efficiently adhere to intestinal epithelial cells. It must also obey other technical qualities such as auto-aggregation, co-aggregation, cell surface hydrophobicity, vigorous antimicrobial activity against enteric pathogens, and boosting the immune response (Saadat et al., 2019). Antioxidant and anticancer properties provide a potential platform for their therapeutic applications (Barigela and Bhukya, 2021). The influence of LAB against enteric pathogens, reducing toxicity and increasing nutritive value of fermented foods has been studied by Bartkiene et al. (2018). Numerous probiotic studies have shown promising results in improving health ailments such as anxiety, depression (Aslam et al., 2020), antibiotic-related diarrhea, irritable bowel syndrome (Sanders et al., 2019), and alleviation of lactose intolerance (Aspri et al., 2020).

The prime objective of the current study is to gain awareness and explore the nutritional and probiotic qualities of TPN. Selected LAB were analyzed for their *in vitro* probiotic attributes to confirm their potential health benefits and therapeutic applications.

## Materials and Methods

### Sample Collection and Processing

Twenty-six fresh samples of toddy palm nectar (TPN) were collected from different geographical areas of Telangana, India in sterilized polythene bags under hygienic conditions and brought to the laboratory in an icebox at 4°C, without exposure to the sun. One set of samples was directly stored in the refrigerator and another set of samples were centrifuged at 10,000 rpm for 10 min, supernatants were filtered through a 0.22 μm membrane filter (Sartorius AG, Göttingen, Germany) and stored at –20°C for further analysis. The pH of TPN samples was determined using a pH meter (Systronics, India).

### Chemical Profile of Toddy Palm Nectar

#### Evaluation of Total Sugars and Ethanol by HPLC

Total sugars and ethanol from fresh TPN were analyzed on HPLC (Shimadzu Inc.) with the method described by Lefebvre et al., 2002. Analysis was performed under isocratic conditions using a platinum amino column (250 × 4.6 mm, 5 μm) and a photodiode array detector (PDA) maintained at 40°C. The mobile phase consisted of 0.001 N H_2_SO_4_ was pumped with a flow rate of 1 ml/min with an injection volume of 20 μl. The eluent and samples were filtered using 0.22 μm nylon membrane filters (Sartorius AG, Göttingen, Germany) prior to analysis. All chemicals used were of HPLC grade and supplied by Sigma-Aldrich. Elution was monitored at 215 nm. Ethanol and different sugars like sucrose, fructose, glucose, galactose, lactose, arabinose, raffinose were identified by comparing the retention times with authentic standards and their concentrations were determined.

#### Evaluation of Volatile Fatty Acids by GC-MS Analysis

Gas chromatography and mass spectrometry (GC-MS) analysis were carried out as described by Dos Santos et al., 2011 using GCMS-QP2010 PLUS (Shimadzu, Japan) with DB5 MS (0.25 × 30 × 0.25) column. Helium (carrier) was used at a rate of 1 ml/min and an injection volume of 1μl was used with an injector temperature of 280°C and ion-source temperature of 200°C. The temperature was maintained at 100°C (isothermal for 4 min), with an increase of 10°C/min, up to 200°C. The temperature was then raised at a level of 4°C until it reached 280°C and maintained for 12.95 min. The sample was prepared by mixing 500 μl of TPN with 200 μl chloroform, vortexed for 5 min and allowed to settle, followed by centrifugation at 4,000 rpm for 10 min. The chloroform layer was collected, and the above step was repeated three times After each step, chloroform layers were pooled and dried under vacuum. Derivatization was carried out for the speed vac dried pellet with methanol and heated at 60°C for 20 min. Subsequently, 1 ml of chloroform was added and after a short spin, approximately 100 μl of chloroform layer was taken for injection. Mass spectra were recorded at two scans per second with a scanning interval of 50 –600 m/z. Compounds were identified based on GC retention times and compared with standard mass spectra using the Wiley and NIST (National Institute of Standards and Technology) Librarie 11.

#### Estimation and Quantification of Amino Acids by Ultra-Performance Liquid Chromatography (UPLC)

Amino acid profiling was carried out by UPLC (Waters Acquity) equipped with a PDA detector according to the previous protocol (Szkudzińska et al., 2017). The column temperature was maintained at 55°C and 260 nm wavelength with a flow rate of 0.7 ml/min. In brief, 2 ml of 6 M HCL was taken into a 50 ml flat bottom tube containing internal standard. Hundred microliter of the test sample was taken into a separate clean glass vial and inserted into a flat bottom tube. The tube was sealed with parafilm and placed in a dry bath at 60°C under N_2_ gas for 15 min, to maintain inertness. The temperature of the dry bath was gradually increased to 110°C and the incubation was extended till 24 h to get the pellet. Borate buffer (200 μl) was added to the pellet, vortexed, and centrifuged. Later 10 μl of sample from the supernatant was added with 70 μl of borate buffer and 20 μl of Accq Tag ultra-reagent and incubated for 10 min at 55°C for derivatization. After incubation, 1 μl was loaded into UPLC and quantified using amino acid standards (Sigma).

#### Isolation and Screening of LAB From TPN Samples

From 26 TPN samples, 1 ml each was added to de Man, Rogosa, and Sharpe (MRS) broth and incubated at 37°C for 24 h in aerobic conditions, and a further 10- fold dilution was made up to 10^–7^ by adding phosphate buffer saline (PBS). Aliquots of 0.1 ml of each dilution were seeded on MRS agar plates containing CaCO_3_ by the spread plate method (Xiao et al., 2015) to distinguish acid-producing bacteria. The bacterial colonies forming clear zones due to the hydrolysis of CaCO_3_ around them were considered as LAB and were individually picked and streaked on MRS agar for further screening. After incubation, 120 morphologically discrete colonies were randomly selected, obtained LAB isolates were subcultured on MRS agar plates and stored at 4°C for further characterization. All LAB isolates were examined by Gram staining, Catalase test, their tolerance to salts and temperature was evaluated and morphology was studied by microscopic observation. Isolates were cultured at different temperatures (10–45°C) and different concentrations of NaCl (2, 4, 6% w/v) (Ni et al., 2015).

#### Molecular Identification of LAB Isolates

Extraction of bacterial genomic DNA was done using the MagGenome XpressDNA isolation kit (India). Amplification of 16S rRNA was performed using a primer set of 27F (5AGAGTTTGAYCCTGGCTCAG-3′) and 1492R (5′-GGCTACCTTGTTACGACTT-3′) (Macrogen, South Korea). Obtained sequences were compared with the data available in the GenBank database by NCBI-BLAST (Altschul et al., 1997). The phylogenetic tree was constructed using the Neighbor-joining method with Mega X software (Saitou and Nei, 1987) and sequences were submitted to NCBI.

#### Safety Evaluation

Hemolytic, proteolytic and gelatinase activities of TPN microflora was evaluated by inoculating the TPN and overnight grown cultures of LAB isolates in Columbia blood agar (BD, Difco), MRS agar supplemented with 1% skimmed milk powder and MRS agar (Himedia, India) supplemented with 5% (w/v) gelatin (HiMedia, India), respectively, and incubating at 37°C under aerobic conditions for 48 h. After incubation, plates were observed for hemolytic properties viz. α-hemolysis (greenish clear zone around the colony), β-hemolysis (clear zone around the colony) and, γ-hemolysis (no clear zone around the colony) (Yasmin et al., 2020). The presence of a clear zone around the colonies in skimmed milk supplemented medium was considered positive for proteolytic activity (Da Silva et al., 2019). Gelatinase activity was observed in gelatin supplemented medium after 72 h. of incubation and subsequently, plates were kept at 4°C for 4 h to observe the clear zone around the colonies as a positive result (Perin et al., 2014). A multiple tube fermentation test was carried out to check the coliform contamination (Mannapperuma et al., 2011). TPN samples were inoculated, incubated at 37°C aerobically for 48 h in MacConkey broth (Himedia, India) to activate coliforms. These active cultures were added to the Brilliant Green Lactose Bile broth (Himedia, India) embedded with Durhams tubes, then incubated at 37°C for 48 h. Coliforms can be confirmed by observing gas production in inverted Durhams tubes and the appearance of a red ring after adding 0.2 ml of Kovac’s reagent to the Brilliant Green Lactose Bile broth.

#### Evaluation of Probiotic Properties

LAB isolates were evaluated *in vitro* for their probiotic potential.

#### Acid and Bile Tolerance

Tolerance to acidic pH was carried out with the methodology described by Zommiti et al. (2018). The overnight grown cultures of LAB isolates were inoculated in MRS broth with pH adjusted to 2.5 and 3.0 using 1N Hydrochloric acid (HCL) and incubated aerobically at 37°C for 3 h. The MRS broth with initial pH of 6.5 was considered as a control for acidic pH comparison. Samples were collected at every 1 h interval to check for the viability of isolates for 3 h and calculated the log cfu/ml. Bile salt tolerance of isolates was carried out using the protocol of de Albuquerque et al. (2018). The overnight grown LAB cultures were inoculated in MRS broth supplemented with 0.3% (w/v) Ox-gall and incubated at 37°C for 4 h. MRS broth without bile salt was used as a control for comparison. Samples were collected at every 1h interval, serially diluted, plated on MRS agar, and incubated at 37°C for 48 h and viable cell counts (log cfu/ml) were determined.

#### Survival in Simulated Gastric Juice

Subsequently, the selected isolates from each of the above experiments were further tested to determine their ability to survive in a gastric environment by inoculating them in simulated gastric juice as described by Singhal et al., 2019. Five ml of overnight grown cultures of LAB strains were centrifuged, and the bacterial pellet was washed and re-suspended in 4 ml of saline (0.8% NaCl). To the 1 ml of cell suspension, 9 ml of simulated gastric juice (pH 3) was added and vortexed for 15 s. After incubation for 1, 2, and 3 h, cells were harvested by taking 1 ml of sample broth into sterile 2 ml Eppendorf tube, then centrifuged at 10,000 rpm for 5 min. Viable counts were determined by growing on MRS agar incubated at 37°C for 24 h. Cell viability (log cfu/ml) was assessed using the plate count method. MRS medium without simulated gastric juice was used as a control. Survival (%) of the organisms was calculated as follows: % Survival = (log no of viable cells survived/log no of initially viable cells) × 100.

#### Auto Aggregation, Coaggregation and Cell Surface Hydrophobicity

Auto aggregation and coaggregation ability of LAB isolates were carried out by the method of Choi et al. (2018). For autoaggregation, overnight grown LAB cultures were harvested, washed, and re-suspended in PBS. The optical density of suspension was adjusted to 0.50 at 600 nm (OD_600_) and incubated aerobically at 37°C without agitation. OD_600_ was measured after 24 h of incubation, and the percent aggregation was determined as follows: A% = (1– At/A_0_) ×100, where A0 and At refers to the OD_600_ at 0 h and at the indicated time, respectively.

The ability of LAB isolates to aggregate with enteric pathogens like *Escherichia coli* MTCC 452, *Salmonella enterica* ser. *paratyphi* MTCC 3216, *Enterococcus faecalis* MTCC 6845, *Proteus vulgaris* MTCC426, and *Klebsiella pneumoniae* MTCC 109 was studied. Equal volumes of the LAB cultures and selected pathogenic bacteria were mixed after adjusting the OD_600_ to 0.5. The co-aggregation was expressed as the percentage reduction in the absorbance of the mixed suspension compared to the individual suspensions by taking the OD_600_ after 6 and 24 h incubation. The growth rate of pathogenic bacteria without adding cell-free supernatant (CFS) was considered as 100% (control).

Further, LAB isolates were also assessed for cell surface hydrophobicity, by measuring adhesion capacity to hydrocarbons (Rokana et al., 2018). Overnight cultures were harvested by centrifugation at 8,000 rpm, 4°C for 10 min, and the pellet was washed twice with PBS and re-suspended in the same buffer followed by measurement of OD_600_. Three milliliter of cell suspension was blended with 1 ml of hydrocarbon (xylene) and incubated without shaking at 37°C for 1 h to get aqueous and organic phases separately. One milliliter of the aqueous phase was removed carefully and the OD_600_ was measured. Cell surface hydrophobicity was calculated using the following formula: Cell surface hydrophobicity % = (1– A_1_/A_0_] × 100.

#### Antibiotic Sensitivity

The antibiotic sensitivity of selected LAB isolates was assessed by the disc diffusion method (Singhal et al., 2019). Antibiotic discs of Penicillin-G (10 units), ampicillin (AMP 10 μg), polymyxin-B (300 units), vancomycin (VA 30 μg), amoxicillin (AMX 10 μg), rifampicin (RIF 5 μg), trimethoprim (TR 10μg), norfloxacin (NX 10 μg), ciprofloxacin (CIP 5 μg), streptomycin (S 10 μg), chloramphenicol (C 30 μg), tetracycline (TE 30 μg), clindamycin (CD 2 μg), erythromycin (E 15 μg), lincomycin (L 10 μg), kanamycin (K 30 μg) and gentamycin (GEN 10 μg) were chosen based on the recommendations of European Security Food Authority (EFSA Panel on Additives and Products or Substances used in Animal Feed (FEEDAP), 2012). Hundred microliter of overnight grown cultures of LAB were spread onto the MRS medium plates, allowed to dry and antibiotic discs were placed, then incubated aerobically at 37°C for 24 h. The diameter of the clear zone of inhibition was measured using an antibiotic zone scale. Results obtained were expressed as sensitivity/resistance in mm.

#### Antimicrobial Activity Against Enteric Pathogens

The antimicrobial feature of LAB isolates against enteric pathogens was assessed using the agar well diffusion method (Yadav et al., 2016). Indicator organisms like *Escherichia coli* MTCC452, *Salmonella enterica* ser. *paratyphi* MTCC 3216, *Pseudomonas aeruginosa* MTCC424, *Enterococcus faecalis* MTCC 6845, *Proteus vulgaris* MTCC426, *and Klebsiella pneumoniae* MTCC109 were incubated in Luria-Bertani broth for 24 h, diluted until it gets the OD_600_ of 0.06, then spread on Muller Hinton agar. Neutralized cell-free supernatant (nCFS) was prepared by centrifuging overnight cultures of LAB isolates at 10,000 × g/10 min at 4°C, and the pH of the supernatant was adjusted to 6.5 using 5 M NaOH. Another set of CFS without neutralization was also prepared to differentiate the impact of pH. Subsequently, 30 μl of cell-free supernatant (CFS) of LAB cultures were placed in each well and incubated for 24 h at 37°C to determine the zone of inhibition around the well.

#### Antioxidant Assay

DPPH radical scavenging ability of LAB was assessed by the method described by Xing et al. (2015). Briefly, 100 μl of freshly prepared 0.2 mM DPPH solution (in methanol) was added to 96- well plate comprising different concentrations of sample, then made up to 200 μl with distilled water. The sample was vortexed and kept at 37°C for 30 min in dark. Blank was prepared by replacing DPPH with methanol. Scavenged DPPH was examined by determining the absorbance at 517 nm against a blank on a microplate reader (Epoch Biotech). Ascorbic acid was used as standard and methanol along with DPPH served as control. IC_50_ values were calculated from the data to find out the concentration of sample required to eliminate DPPH free radicals by 50%. Percentage inhibition to scavenge the DPPH radicals was calculated using the following formula:

DPPH activity (μl/ml) = [(Ao-Ae)/Ao] × 100

where, Ao and Ae are the absorbance of the control and test samples, respectively.

#### Cell Culture and Adhesion Assay

HT-29 cell lines (human colon adenocarcinoma) were obtained from National Cell Repository at NCCS (Pune, India) and maintained in a Minimum Essential Medium (MEM) supplemented with 20% heat-inactivated Fetal Bovine Serum (FBS) and penicillin 10 U/ml, then incubated at 37°C in a 5% CO_2_ incubator. Adhesion of LAB cells to HT-29 cells was carried out by the method described by Sharma and Kanwar (2017). HT-29 cells (5 × 10^5^ cells/ml) were seeded in a six-well plate and cultured until the cells reached the required confluence. Overnight cultures of LAB isolates were harvested, washed twice, and re-suspended in antibiotic-free Dulbecco’s Modified Eagle Medium (DMEM) at a concentration of 10^9^ cfu/ml and added to HT-29 cells. Later 300 μl of methanol was added to each well, followed by incubation for 10 min at room temperature. Methanol was completely removed and cells were fixed by Giemsa staining (0.72% w/v; Sigma) for 30 min at room temperature. Plates were washed with ethanol, air-dried, and bacterial adhesion was examined under an inverted microscope (Olympus BX64, Japan) at a scale of 200 μm.

#### Anticancer Activity by MTT [-(4, 5-Dimethylthiazol- 2-yl)-2, 5-Diphenyltetrazolium Bromide] Assay

Effect of CFS of LAB on HT-29 colon cancer cell lines for anti-proliferation ability was evaluated by MTT assay according to the protocol of Chandel et al. (2019). Each well of the 96-well plate was seeded with 5 × 10^5^ cells in 200 μl MEM. After 24 h, CFS of LAB were added to each well in a volume-dependent mode (20, 40, 60, 80, and 100 μl) and incubated for 24 h. After precise incubation, 50 μl of MTT solution (0.4 mg/ml) was added to each well and re-incubated for 4 h in a 5% CO_2_ incubator. After incubation, the MTT solution was replaced with 100 μl of dimethyl sulfoxide to solubilize the formazan crystals and incubated for 30 min at 37°C. The absorbance of each well was measured using an ELISA reader (Epoch Biotech microplate reader) at 570 nm. Results were expressed as % anti-cancer activity of LAB which was calculated as 1-(OD of test sample/OD of control)X 100. For comparison, MRS broth was taken as control. IC_50_ values were calculated to know the required CFS of LAB to obtain 50% anti-cancer activity.

#### Detection of Cell Apoptosis by DAPI (4′, 6-Diamidino- 2-Phenylindole) Staining

Two ml of HT-29 cells (1.2 × 10^5^ cells/ml) were added to each well of a six-well plate and incubated in a 5% CO_2_ incubator at 37°C for 48 h. After achieving a confluence of 50 –60%, 500 μl of selected LAB-CFS was added to each well, and wells without CFS were considered as control. After 24 h, cells were carefully washed with DMEM and then 4% formaldehyde was added. After 5 min of incubation, the fixed cells were washed twice with PBS, permeabilized, and then again treated with PBS containing 0.1% Triton X-100 for 5 min at 37°C. Cells were stained with 50 μl of DNA-intercalating agent DAPI (1:2,000 dilution) and incubated for 24 h at 37°C (Nami et al., 2015). Subsequently, the plates were washed with PBS and evaluated under an inverted microscope with a U-MWU2 fluorescence filter (Olympus BX64, Japan).

#### Statistical Analysis

Each experiment was carried out in triplicates. Values were statistically analyzed and expressed as mean ± standard deviation (SD). Significant differences in the results of each test were determined by comparing relative control values by ANOVA (Analysis of Variance) using Graph Pad Prism software. *P* < *0.05* was considered statistically significant.

## Results

### Chemical Analysis of Palm Nectar

#### Sugar, Ethanol, Fatty Acid, and Amino Acid Profile

To understand the nutritional and therapeutic efficiency of TPN, sugar, ethanol, fatty acid (VFA), and amino acid profiling was done. Among sugars, glucose, arabinose, and galactose were detected at retention time (RT) 6.3, 6.4, and 6.2, respectively. Glucose was found to be the highest with 4.37 mg/ml and arabinose was the least with 2.85 mg/ml. The average ethanol concentration of fresh TPN was found to be 0.3 mg/ml. A total of 18 major VFAs were identified in the crude TPN chloroform extract on GC-MS. Among them, significant peaks were observed for hexadecenoic acid, methyl palmitate at RT 23.86 (M/Z 74), E-15-heptadecenal at RT 21.17 (M/Z55) and E-14-hexadecenal at RT 16.73(M/Z55) (Table 1). Similarly, a total of 26 primary amino acids were identified in fresh TPN. OH-lysine-2 (12.86% mole) was being the major one and alanine (12.52% mole) and leucine (6.08% mole) were identified as the second and third major amino acids, respectively, while histidine (0.30% mole) was the lowest one (Table 2).

**TABLE 1:** Volatile fatty acid composition of toddy palm nectar detected by GC-MS (gas chromatography and mass spectrometry).

Name of the compound	RT	M/Z	Area	Biological importance
Triethyl ester (CAS) Boron ethoxide	1.578	73	5375199	Anti-microbial
Butanedioic acid, dimethyl ester	3.296	115	2810517	Flavoring agent
Dodecane, 2,6,11-trimethyl-	3.773	57	1598524	Anti-microbial
1-Dodecene (CAS) Adacene 12	6.62	55	2002846	Anti-bacterial
Permethylated and reduced globicide	4.607	115	896593	Anti-coagulant
Benzene, 1,3-bis(1,1-dimethylethyl)-	8.268	175	5198754	Adenocarcinoma
Benzaldehyde, 4-propyl-	8.755	91	1669626	Biological applications
Dodecane, 2,6,11-trimethyl-	8.901	57	922289	Anti-bacterial
1-Non-adecene (CAS)	9.808	69	333589	Anti-cancer
Hexadecane, 2,6,11,15-tetramethyl-	10.1	57	1879178	Flavoring agent
Tetratriacontane (CAS) n-	12.58	57	714879	Anti-cancer
Eicosane	14.43	57	5924526	Anti-bacterial
Eicosanoic acid, Arachidic acid	15.09	74	1879512	Anti-oxidant
E-14-Hexadecenal	16.73	55	6683918	Anti-inflammatory
Octadecane (CAS) n-Octadecane	19.42	57	5421797	Role in Pheramones
2-Butoxysulfonylhexadecane	20.35	57	1718274	Anti-microbial
E-15-Heptadecenal	21.17	55	7676234	Anti-oxidant
Hexadecanoic acid, Palmitic acid	23.86	74	11666977	Anti-oxidant

**TABLE 2:** Amino acids profile from toddy palm nectar detected by UPLC (ultra-performance liquid chromatography).

Name	Conc in μ g/ml	%mole	Biological importance
Histidine	246.74	0.302	Protein interactions, Precursor of histamine
Aspargine	242.38	0.348	Protein synthesis
Serine	1566.74	2.833	Biosynthesis of purines and pyrimidines
Arginine	1275.58	1.391	Cardiovascular diseases, anti-aging
Glycine	692.69	1.754	Schizophrenia, anti-inflammatory
Aspartic acid	1248.02	1.782	Vital role in neuro endocrine system.
Citruline	3214.16	3.487	Alzheimer’s, dementia, Sickle cell
Glutamic acid	2586.01	3.341	Important neurotransmitter
Sarcosine	1351.66	2.884	Used as adjunctive therapy in Schizophrenia
Threonine	2415.59	3.855	Immunostimulant, better livestock growth
Alanine	5870.45	12.52	Treat Hypoglycemia, prostatic hypertrophy
GABA	1722.08	3.175	Anxiety and improves mood, PMS
aAAA	617.403	0.7281	–
bAIBA	1156.95	2.132	Expression of brown adipocyte
Proline	1165.84	1.924	Proteinogenic
OH-Lysine-1	1573.41	1.844	Collagen formation
OH-Lysine-2	10974.76	12.86	Multifunctional enzyme
Ornithine	1253.58	1.802	Reduces stress and fatigue
Cystine	660.55	1.036	Antioxidant and anti-aging
Lysine	3797.71	4.937	Calcium absorption; proteinogenic
Tyrosine	3489.97	3.661	Stress reliever
Methionine	745.49	0.949	Growth and tissue repair.
Valine	2554.57	4.143	Improves dendritic cell function
Leucine	4202.03	6.088	Growth hormone production
Phenylalanine	2318.24	2.667	Neuro transmitter, anti-depressant
Trptophan	388.30	0.361	Sleep aid and psychiatric disorders

#### Bacterial Isolation and Characterization

**FIGURE 1:**
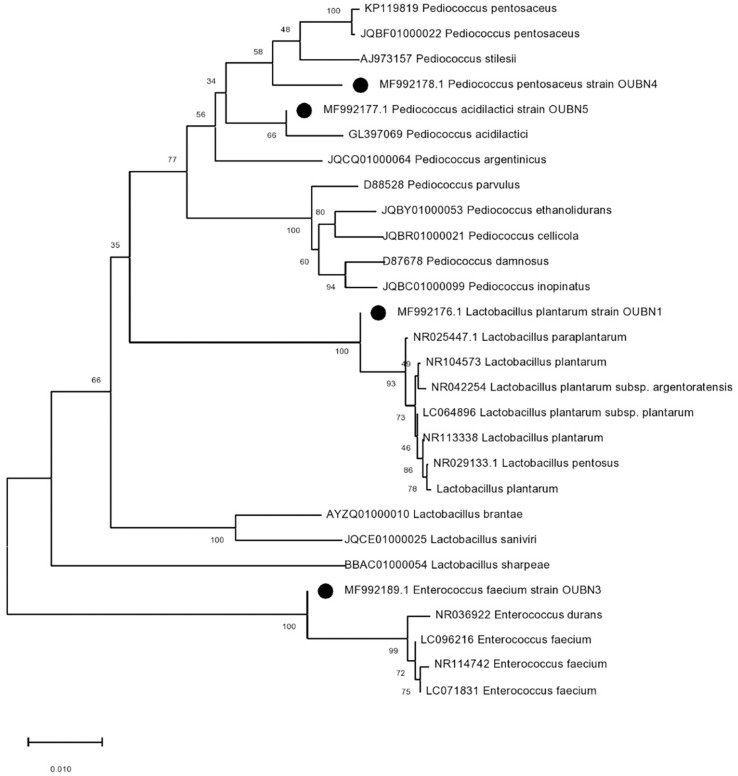
Phylogenetic tree constructed by the neighbour-joining method showing the relative positions of LAB isolates (OUBN1, OUBN3, OUBN4, and OUBN5) based on 16S rRNA gene sequences from Palm toddy nectar samples. Evolutionary analyses were conducted in MEGA X. The tree is drawn to scale. The percentage of replicate trees in which the associated taxa are clustered together in the bootstrap test of 1,000 replicates is shown next to the branches.

A total of 120 bacterial isolates were obtained from twenty-six TPN samples on MRS agar with CaCO_3_ then further subjected to physiological and biochemical screening. Gram-positive and catalase-negative isolates were presumptively identified as LAB. The growth of four selected isolates at various salinity and temperatures showed that the isolates displayed the ability to grow in presence of 6% NaCl and at 42°C except for one isolate OUBN4 (Table 3). Based on the above, these four LAB isolates were further characterized at the species level by 16S rRNA sequencing analysis. Phylogeny of these four LAB isolates viz. OUBN1, OUBN3, OUBN4, and OUBN5 have shown maximum similarity with the *Lactobacillus plantarum* group (99.18%), *Enterococcus faecium* (99.85%), *Pediococcus acidilactici* (100%), and *Pediococcus pentosaceous* (98.33%), respectively (Figure 1). Sequences were submitted to the NCBI GenBank database and accession numbers were obtained for the gene sequences of isolate OUBN1 (MF992176), OUBN3 (MF992189), OUBN4 (MF992177), and OUBN5 (MF992178).

**TABLE 3:** Morphological, biochemical, and physiological characteristics of the LAB Isolates from Toddy palm nectar.

Characteristics	OUBN1	OUBN3	OUBN4	OUBN5
Shape	Rod	Cocci	Cocci	Cocci
Catalase activity	–	–	–	–
Growth in 0.5% NaCl	+	+	+	+
1%NaCl	+	+	+	+
2%NaCl	+	+	+	+
4%NaCl	+	+	+	+
6%NaCl	+	+	-	+
**Growth At**				
20°C	+	+	+	+
30°C	+	+	+	+
37°C	+	+	+	+
40°C	+	+	+	+
42°C	+	+	–	+

**Positive results (+), Negative results (–).**

#### Safety Evaluation

TPN samples were analyzed for their microbiological safety to confirm their plausibility as a safe drink. In our study, TPN samples and four selected strains showed non-hemolytic, non-gelatinase, and non-proteolytic activity, indicating that the TPN and micro-organisms present in TPN are non-pathogenic. Results of the presumptive coliform test also revealed the absence of coliforms in the TPN samples.

### Evaluation of Probiotic Properties

#### Survival in Acidic pH, Bile, and Simulated Gastric Juice

The tolerance of isolates to acidic pH shows their ability to survive in hostile conditions of the gastrointestinal tract. Selected LAB isolates showed maximum survival at pH 2.5 –3.0 at 37°C for 3 h (Table 4A). Tolerance to bile salts helps in evaluating the ability of isolates for their establishment in the gastric environment. All 4 isolates showed maximum survival with 0.3% ox bile after 1-3 h of treatment at 37°C (Table 4B). All isolates were found to maintain above 95% viability (*P* < 0.001) at pH 3 and have shown good viable count after 3 h exposure to 0.3% bile concentration (*P* < 0.001). Further, all isolates survived well in simulated gastric juice with above 90% viability after 1-3 h of incubation at 37°C. In particular, isolates OUBN3 and OUBN5 showed the highest viability of 8.94 log cfu/ml at 3 h incubation (Table 4C).

**TABLE 4A:** Viable cell count log cfu/ml of LAB survived in MRS broth at different pH and time intervals.

LAB strains	pH 2.5				pH 3.0			
		
	0 h	1 h	2 h	3 h	0 h	1 h	2 h	3 h
OUBN1	7.01 ± 0.05	6.67 ± 0.04	6.62 ± 0.01	6.53 ± 0.04	6.93 ± 0.01	6.85 ± 0.02	6.78 ± 0.05	6.72 ± 0.05
OUBN3	6.98 ± 0.04	6.50 ± 0.04	6.40 ± 0.05	6.91 ± 0.04	6.90 ± 0.04	6.89 ± 0.04	6.86 ± 0.04	6.84 ± 0.05
OUBN4	7.05 ± 0.03	6.85 ± 0.02	6.81 ± 0.01	6.75 ± 0.03	6.96 ± 0.03	6.90 ± 0.05	6.85 ± 0.04	6.89 ± 0.02
OUBN5	7.03 ± 0.03	6.59 ± 0.03	6.56 ± 0.02	6.51 ± 0.02	6.99 ± 0.03	6.96 ± 0.03	6.88 ± 0.03	6.94 ± 0.02

**The mean of three values is presented ± SD for each sample. Viable counts were expressed in log10 cfu/ml. P-value is significant (P < 0.001).**

**TABLE 4B:** Viable cell count log cfu/ml of LAB survived in MRS after 0, 1, 3, and 4 h in the presence of 0.3% bile salts.

	Viable cell count log cfu/ml				
	
LAB strains	0 h	1 h	2 h	3 h	4 h
OUBN1	7.90 ± 0.03	7.67 ± 0.05	7.78 ± 0.05	7.84 ± 0.05	7.85 ± 0.03
OUBN3	8.04 ± 0.01	7.71 ± 0.06	7.69 ± 0.07	7.82 ± 0.03	7.81 ± 0.03
OUBN4	7.99 ± 0.03	7.72 ± 0.04	7.84 ± 0.03	7.90 ± 0.02	7.87 ± 0.04
OUBN5	8.01 ± 0.03	7.71 ± 0.03	7.85 ± 0.08	7.98 ± 0.03	7.92 ± 0.03

**The mean of three values is represented as ± SD for each sample. Viable counts were expressed in log10 cfu/ml. P-value is highly significant (P < 0.0001).**

**TABLE 4C:** Viable cell count log cfu/ml of LAB survived in simulated gastric juice at different time intervals.

	Viable cell count log cfu/ml			
	
LAB strains	0 h	1 h	2 h	3 h
OUBN1	6.96 ± 0.05	6.89 ± 0.04	6.88 ± 0.03	6.82 ± 0.03
OUBN3	9.00 ± 0.02	8.94 ± 0.03	8.95 ± 0.02	8.94 ± 0.02
OUBN4	7.98 ± 0.01	7.94 ± 0.02	7.91 ± 0.06	7.90 ± 0.03
OUBN5	9.01 ± 0.03	8.93 ± 0.03	8.92 ± 0.05	8.94 ± 0.03

**The mean of three values is presented for each sample ± SD. Viable counts wer expressed in log cfu/ml. No significant (P < 0.17) difference in the viability of the cells in the gastric conditions incubated for 3 h.**

#### Auto Aggregation, Hydrophobicity, and Coaggregation

**FIGURE 2:**
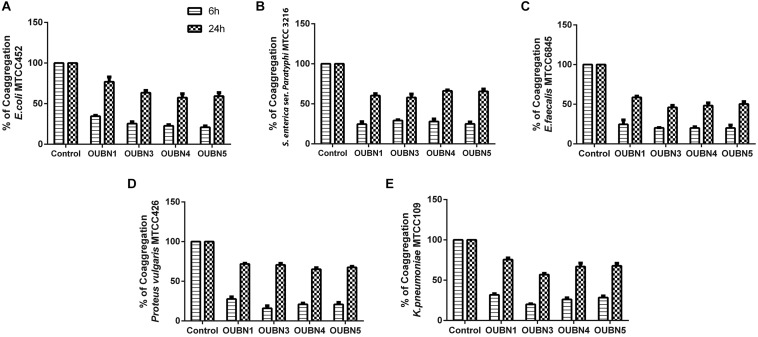
**(A)** Assessment of Auto aggregation and hydrophobicity of LAB isolates. **(B)** Percentage of coaggregation by selected LAB isolates after 6 and 24 h against enteric pathogens **(A)** *Escherichia coli* MTCC 452. **(B)** *Salmonella enterica* ser. *paratyphi* MTCC3216. **(C)** *Enterococcus faecalis* MTCC 6845. **(D)** *Proteus vulgaris* MTCC 426. **(E)** *Klebsiella pneumoniae* MTCC 109. Bacterial growth was monitored at OD_600_. The growth of enteric pathogens without CFS was considered as control (100%). Data shown are mean ± SD of triplicate values of independent experiments. *P* > 0.005.

Isolate OUBN1 has shown higher autoaggregation activity (81.76%), while OUBN3 showed the lowest autoaggregation (69.26%) (Figure 2A). Also, the cell surface hydrophobicity of 79.71% was observed for OUBN1, which was significantly higher (*p* < 0.05) when compared to OUBN5 (70.15%). Whereas, there was no significant difference (*p* > 0.05) in cell surface hydrophobicity of LAB isolates OUBN3 and OUBN4 (Figure 2A). All four LAB isolates had good co-aggregation ability with pathogens after 24 h incubation. Of all these isolates, OUBN1 displayed highest percentage of coaggregation with *E. coli* MTCC452, *K. pneumoniae* MTCC109, and *S. aureus* MTCC902 (Figure 2B).

#### Antibiotic Sensitivity

The antibiotic sensitivity of LAB isolates against different antibiotics was determined and obtained results were equated with the interpretative chart of zone size provided in the catalog (Table 5). The four isolates were highly susceptible to antibiotics such as chloramphenicol, tetracycline, penicillin-G, clindamycin, erythromycin, rifampicin, and lincomycin; while resistant to trimethoprim, ampicillin, streptomycin, polymyxin-B, vancomycin, gentamycin, amoxicillin, norfloxacin, ciprofloxacin, and kanamycin.

**TABLE 5:** Antibiotic susceptibility of LAB strains isolated from toddy palm nectar samples.

	ZOI in mm			
	
Antibiotics	OUBN1	OUBN3	OUBN4	OUBN5
Trimethoprim (10μg)	–	–	+	+
Ampicillin (10 μg)	–	–	–	–
Streptomycin (10 μg)	–	–	–	–
Chloramphenicol (30 μg)	+ +++	+++ +	+++	+++ +
Polymyxin-B (300 units)	–	–	–	–
Tetracycline (30 μg)	+ +++	+++ +	+ +++	+++ +
Penicillin-G (10 units)	+ ++	+++	+ ++	+++
Vancomycin (30 μg)	–	–	–	–
Clindamycin (2 μg)	+ +++	+++ +	+ +++	+++ +
Gentamycin (10 μg)	+	+	–	–
Amoxycillin (10 μg)	+	–	+	+
Erythromycin (15 μg)	+ ++	+++ +	+ +++	
Rifampicin (5 μg)	++++	++++	++++	++++
Norfloxacin (10 μg)	–	–	–	–
Ciprofloxacin (5 μg)	–	–	–	–
Lincomycin (10 μg)	+ +	+++	+ ++	+++
Kanamycin (30 μg)	–	–	–	–

**ZOI (zone of inhibition) - no effect detected, +, ++, +++, ++++ represents diameter of zone of inhibition between 1–2, 2–5, 5–10 and more than 10 mm, respectively.**

#### Antimicrobial Activity of LAB Isolates

The antimicrobial activity of selected LAB isolates against common enteric pathogens was tested. The un-neutralized CFS of all 4 isolates inhibited the growth of all pathogens tested. Whereas, neutralized CFS (nCFS) inhibited the growth of only a few tested pathogens (Table 6). The nCFS of OUBN1 inhibited the growth of *P. aeruginosa* MTCC424 and *P. vulgaris* MTCC426, whereas nCFS of OUBN3 inhibited *E. coli* MTCC 452, *P. aeruginosa* MTCC424, *E. faecalis* MTCC 6845, and *K. pneumoniae* MTCC 109. It was also observed that nCFS of OUBN5 inhibited the growth of *P. aeruginosa* MTCC424 only while un-neutralized CFS and nCFS of OUBN4 inhibited the growth of all pathogens tested in this study.

**TABLE 6:** Antimicrobial activity against enteric pathogens shown by LAB isolated from toddy palm nectar samples

Pathogens	Zone of inhibition in cm							
	
	OUBN1		OUBN3		OUBN4		OUBN5	
				
	CFS	nCFS	CFS	nCFS	CFS	nCFS	CFS	nCFS
*E. coli* MTCC 452	1.8	–	1.6	1.2	1.6	1.4	1.8	–
*P. aeruginosa* MTCC424	1.8	1.0	1.6	1.0	1.8	1.0	1.8	1.2
*P. vulgaris* MTCC426	1.6	0.6	1.8	–	1.6	0.6	1.6	–
*S. para typhi enterica* ser. MTCC 3216	1.4	–	1.4	–	1.4	0.6	1.6	–
*E. faecalis* MTCC 6845	1.6	1.6	1.6	1.6	1.8	1.2	1.6	–
*K. pneumoniae* MTCC 109	2.0	1.6	2.0	1.8	2.4	1.8	2.0	–

**CFS (Cell-free supernatant), nCFS (Neutralized cell-free supernatant).**

#### DPPH Radical Scavenging and Adhesion Activity

**FIGURE 3:**
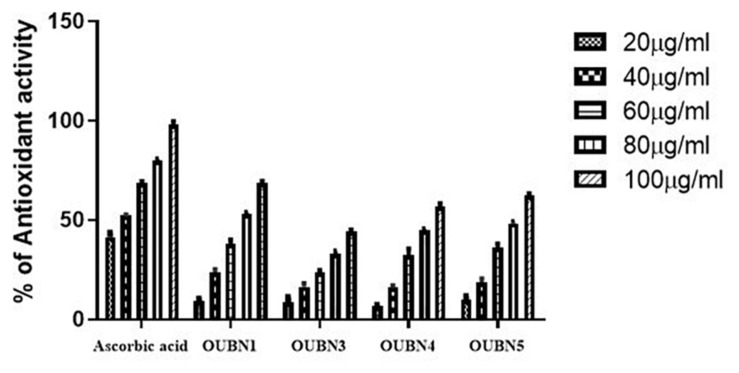
Antioxidant abilities of LAB isolates by using CFS (cell free supernatant) of 20, 40, 60, 80, 100 μg/ml concentrations from Ascorbic acid, OUBN1, OUBN3, OUBN4, and OUBN5, respectively. Ascorbic acid was used as standard.

Radical scavenging activity of LAB isolates ranged from 44.05 to 68.71% and IC_50_ was from 75.62 to 117.11 μg/ml (Figure 3). Among the four isolates, OUBN1 showed significantly higher (*p* < 0.05) hydroxyl radical scavenging activity of 68.71% with an IC_50_ of 75.62 μg/ml, followed by OUBN5 (62.48%) and OUBN4 (56.59%) with IC_50_ of 82.23 and 89.01 μg/ml, respectively. Whereas, OUBN3 was found to have lower scavenging activity of 44.05% with IC_50_ of 117.11 μg/ml compared to other LAB isolates.

**FIGURE 4:**
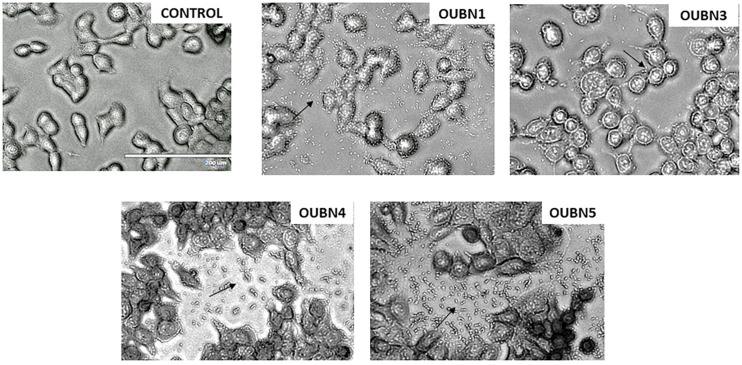
Adhesion of LAB strains on HT-29 cell cultures observed under oil immersion microscope (100X) after staining with Giemsa strain. (Control)- HT-29 cell line without treatment and HT-29 cells treatment with OUBN1, OUBN3, OUBN4, OUBN5, respectively. Scale 200 μm.

Adhesion assay for four LAB isolates was done for their ability to adhere to HT-29 cell lines and considerable variation was observed as presented in Figure 4. Among all isolates, OUBN1 and OUBN5 showed strong adhesion, while OUBN3 and OUBN4 were moderate.

#### Anticancer Activity and Cell Apoptosis

**FIGURE 5:**
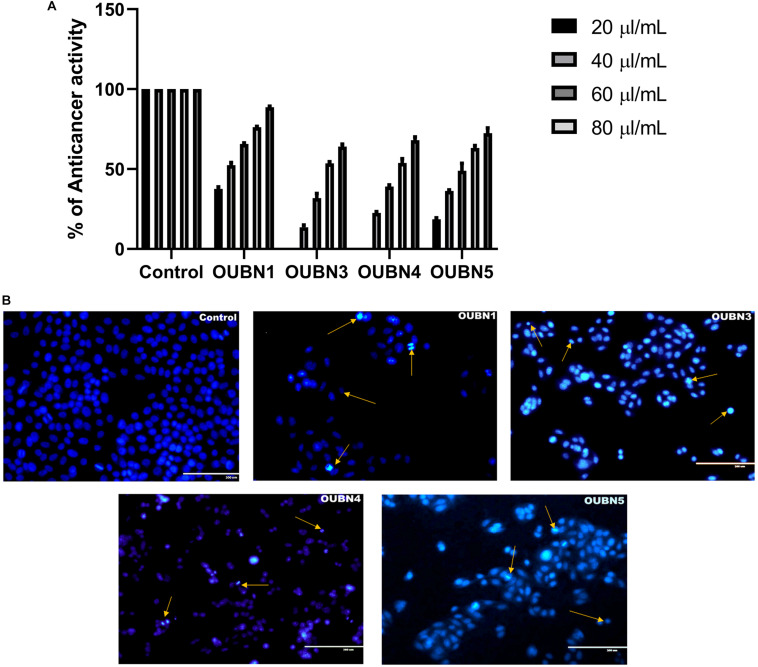
**(A)** Anticancer activity determined by MTT [-(4, 5-Dimethylthiazol-2-yl)-2, 5-diphenyltetrazolium bromide] assay. Experiments were done by using CFS of 20, 40, 60, 80, 100 μl/ml concentrations from CFS of OUBN1, OUBN3, OUBN4, and OUBN5, respectively. Data shown are triplicate values of mean ± SD of independent experiments. **(B)** Apoptosis determined for untreated and treated HT-29 cells with OUBN1, OUBN3, OUBN4, OUBN5 isolates by DAPI (4, 6-diamidino-2-phenylindole) staining. Scale 200 μm.

The CFS of four LAB isolates were tested for their anti-cancer activity against HT-29 cells. Though all four isolates exhibited anti-cancer activity after treatment of cells with 100 μl/ml CFS in contrast to untreated cells, the highest anti-cancer activity of 88.55% was found with isolate OUBN1 while the least activity of 64.05% was found with OUBN3 (Figure 5A).

Subsequently, staining with DAPI was done to detect the visual indication of cell death in untreated and treated cells by fluorescent microscopy. Viable cells were observed to be blue intact, while dead cells were differentiated by bright blue with shrinking and blubbing along with condensed nucleus or fragments that ultimately degenerated, as shown by arrows in Figure 5B.

## Discussion

In the present study, a naturally fermented and easily available drink, toddy palm nectar (TPN) was selected to evaluate its nutritional value and probiotic attributes.

Among the volatile fatty acids (VFA) in such natural ferments, alcohols are the main components and are considered the final standard products of degradation of glucose and amino acid catabolism. Of all VFAs, N-hexadecanoic acid is the major one found in TPN, which is an anti-inflammatory compound and also a phospholipase A2 inhibitor. Hydrolysis of ester bond linkages by phospholipase A2 is the initiating step to start inflammation. Enzyme kinetics study proved that n-hexadecanoic acid inhibits phospholipase A2 in a competitive way (Aparna et al., 2012). Other fatty acids identified include hexadecanoic acid methyl ester (CAS), methyl palmitate, and E-15-heptadecenal, which are responsible for the antibacterial and antioxidant properties of TPN. Several other volatile compounds have been reported previously from different types of palm wines such as *Cocas nucifera* (Borse et al., 2007; Karthikeyan et al., 2014) *Nypa fruticans* (Nur Aimi et al., 2013), and *Elaeis guineensis* (Lasekan and Abbas, 2010). Identification of octadecanoic acid in *Lactobacillus helveticus* by GC–MS was reported by Sharma et al. (2014). Various organic acids and effective antimicrobial activity of *Lactobacillus* strains further supported the organic acid-mediated inhibitory effect (Bajpai et al., 2016). The use of medicinal oils rich in n-hexadecanoic acid for the treatment of rheumatic complications has been described in the traditional medical system of Indian Ayurveda (Aparna et al., 2012). Previous studies confirm the anti-microbial and anti-oxidant properties exerted by the presence of organic acids in fermented foods (Bajpai et al., 2016).

In the present study, 26 amino acids were identified by UPLC, possibly of plant origin, but there is a possibility that microorganisms may also contribute to amino acid production. OH-lysine-2 is an essential amino acid and is found to be the primary amino acid in TPN. It plays a vital role in various biological processes, including the conversion of lipids into metabolic energy, synthesis of collagen fibers, connective tissues, and also participates in the regulation of calcium levels (Peter et al., 2010). Alanine was found as the second major amino acid in TPN tested. Alanine is involved in the metabolism of sugars and acids, which is known to boost immunity and provide energy to the brain, central nervous system, and muscle tissue (Sarah et al., 2013). Leucine was the third major amino acid, essential in protein metabolism, and plays a role in the initiation pathways of muscle protein synthesis. Alanine participates in reversible phosphorylation of proteins that control the binding of mRNA to the 40S ribosomal subunit (Li et al., 2011). GABA (γ-aminobutyric acid) is another important compound found in TPN which is an inhibitory neurotransmitter of the central nervous system. Production of GABA by *L. fermentum* isolated from palm wine was reported by Rayavarapu et al. (2019). Citrulline is also found in TPN and it is the primary precursor of L-arginine in the nitric oxide cycle. Citrulline is known to prevent neuronal cell death and protect against cerebrovascular damage. Therefore, it may provide a neuroprotective role to improve cerebrovascular dysfunction (Lee and Kang, 2018).

Of 26 samples, 120 morphologically different LAB were identified based on differential growth and *in vitro* probiotic characteristics. Previous studies have specified the presence of various LAB, non-LAB, and yeast in palm wine (Tapsoba et al., 2016; Astudillo-Melgar et al., 2019). In our preliminary studies, it was observed that LAB were more abundant than non-LAB and yeasts in TPN. One of our previous studies reported the functional probiotic and therapeutic potential of *Saccharomyces cerevisiae*, which was isolated from palm nectar (Srinivas et al., 2017). Tolerance of selected LAB isolates to various salinity and temperature ranges show that these LAB can endure growing in severe unfavorable environments which is an optimistic feature to select a probiotic.

Based on the above observations, four potential bacterial isolates were selected and subjected to 16S rRNA analysis, confirmed as LAB at the species level, and named as *Lactobacillus plantarum* group OUBN1, *Enterococcus faecium* OUBN3, *Pediococcus acidilactici* OUBN4, and *Pediococcus pentosaceo*us OUBN5. The 16S rRNA analysis revealed high efficacy and congruency for LAB species and selected LAB isolates may be candidates for further investigation as better probiotic strains.

The evaluation of hemolytic activity is regarded as a safety asset according to the European Food Safety Authority (EFSA), to consider the probiotic strains (Joint FAO/WHO Expert Committee on Food Additives Meeting and World Health Organization, 2006). Hemolytic activity of selected strains was evaluated on Columbia blood agar plates and the tested strains showed neither α- hemolytic nor β-hemolytic activity. Our findings were in agreement with the results of Oh and Jung (2015) and Wang et al. (2018), who evaluated the *Lactobacillus* species isolated from millet-based alcoholic beverages fermented by traditional methods and spontaneously fermented non-dairy foodstuffs for their hemolytic activity. Non-hemolytic activity is noteworthy during the selection of probiotic strains, as such strains are non-virulent and lack of hemolysin ensures their non-pathogenic nature (Joint FAO/WHO Expert Committee on Food Additives Meeting and World Health Organization, 2006). Gelatinase enzyme is considered a virulence factor as it may hydrolyze collagens that initiate an inflammatory response (Da Silva et al., 2019). However, in the present investigation, all the LAB were non-hemolytic and non-gelatinolytic. Non-hemolytic and non-gelatinase criterion is measured to be significant to use as starter cultures in the dairy industry (Marroki and Bousmaha-Marroki, 2014). None of the LAB of the present study is positive for proteolytic activity. No pathogenic organisms were detected in any of the toddy (TPN) samples studied, indicating the hygienic status of the tappers, the extraction method involved and the materials used. Results of, non-hemolytic, non-gelatinase, non-proteolytic properties, absence of coliforms, mild ethanol content of TPN indicate its microbiological safe nature.

Tolerance to low pH and high concentration of bile salts is one of the prerequisites for characterizing probiotic strains. Also, they should effectively pass through the gastric stomach and also remain in the small intestine (Anandharaj et al., 2015). Ilavenil et al. (2016) studied four LAB strains and reported a low pH (2.5) and bile salts (0.3%) tolerance, reflecting high survival and proliferation efficiency in hostile intestinal conditions. In our study, exposure to pH 2.5 dramatically reduced the count of different LAB isolates after 2 h of incubation, while efficient growth was observed after 3 h of exposure to pH 3.0. Overall, no significant reduction in CFU count was observed after exposure to pH 3.0. This may be due to a sudden drop in optimal pH and subsequent adaptation to harsh acidic conditions, which has been observed with a higher survival rate in our LAB isolates. Tolerance to 0.3% bile salts was a positive observation with selected LAB isolates which promote easy colonization to the host gut as previously described (Adesulu-Dahunsi et al., 2018). Few studies reported high survival rates of selected LAB strains at pH 2.5 and 0.3% (w/v) bile salts (Nami et al., 2019). Growth of LAB isolates has also been observed in the simulated gastric juice to assess their survival in hostile environments of the gastrointestinal tract. Isolates were able to resist simulated digestive tract conditions with a slight reduction in the number of viable cells, and these results are in agreement with previous findings of Nami et al. (2019).

After entering the intestine, probiotics must adhere to the intestinal mucosa, which accelerates the transient colonization and hinders its elimination by peristalsis. The current study showed a high auto-aggregation of selected LAB isolates, which is in agreement with observations of Somashekaraiah et al. (2019). There is a strong association between auto-aggregation of probiotic strains and their ability to adhere to intestinal epithelial cells, signifying host defense, which is a prerequisite for effective colonization and better persistence in the gastrointestinal tract (Zommiti et al., 2018). Cell surface hydrophobicity has been carried out to determine the efficiency of strains to adhere to the gut region since it is one of the characteristic features of potential probiotic strains. In the present study, four isolates exhibited hydrophobicity greater than 70% and interestingly, strain OUBN1 showed the highest of 79.71%. The auto-aggregation and adhesion efficiency of our isolates are superior to the LAB strains isolated from plant-based fermented food and neera (Choi et al., 2018; Somashekaraiah et al., 2019). Co-aggregation ability of the strains could be a key factor, potentially inhibiting the adherence of pathogenic bacteria to the epithelial surface, which leads to hampering of surface colonization by pathogens. The Auto-aggregation ability of LAB plays a vital role in adhesion to intestinal epithelial cells and thus further prevents the colonization of pathogens. Among the strains, OUBN1 exhibited a maximum coaggregation of > 70% with *E. coli* MTCC452, *K. pneumoniae* MTCC109, and *S. aureus* MTCC902. The inhibitory impact of LAB strains can be associated with the co-aggregation of foodborne pathogenic bacteria. Since the auto-aggregation potential of LAB strains plays an important role in adhesion to intestinal epithelial cells, they further prevent the colonization of pathogens (Yadav et al., 2016). These results proved that the LAB strains assessed in the current study could tolerate and survive efficiently in the human intestinal environment.

To be accepted as an excellent probiotic, organisms must meet specific functional properties like sensitivity to antibiotics and antimicrobial activity against pathogens. The inherent resistance of LAB strains to antibiotics is not considered a risk to human and animal health but may promote therapeutic and preventive benefits when administered together with antibiotics (Al Kassaa et al., 2014). In our study, four LAB isolates were tested for sensitivity to 17 different types of antibiotics and found that they are susceptible to a specific group of antibiotics, ampicillin, erythromycin, chloramphenicol, and tetracycline. Present results are in agreement with the previous study by Caggia et al. (2015) who isolated LAB from kimchi and found it susceptible to penicillin G, erythromycin, and clindamycin which bind to ribosomes, further block protein synthesis, and are effective against Gram-positive microorganisms (Reuben et al., 2020). The LAB isolates of the present study showed resistance to aminoglycosides (gentamicin and streptomycin) sulfonamide (trimethoprim) and glycopeptide (vancomycin) has been reported in LAB, which is associated with its intrinsic resistance resulting from the permeability of its membrane, probably through a resistance flow mechanism that is not transferable (Gueimonde et al., 2013). Furthermore, the electron transport mediated by cytochrome responsible for the absorption of the drug is absent in most of the LAB (Monteagudo-Mera et al., 2012). The intrinsic resistance of LAB strains to antibiotics is not considered a risk to animal and human health but may promote therapeutic and preventive benefits when administered together with antibiotics (Al Kassaa et al., 2014). Natural resistance to ciprofloxacin has been observed in our study, which is consistent with the study conducted by Tang et al. (2018). Geographic location and source of LAB also determined the antibiotic susceptibility patterns of potential probiotic strains (Anandharaj et al., 2015). Antibiotic resistance of bacteria has progressively become an alarming medical problem. Multiple-drug resistance of pathogenic microorganisms against prophylactic antibiotics has become a risk and serious challenge to overcome with the purpose to treat infected persons. Therefore, susceptibility to antibiotics signifies a fundamental prerequisite for probiotics.

Antagonistic activity and production of antimicrobial compounds by LAB against enteric pathogens can be considered as the main probiotic attribute for maintaining the stability of intestinal microbiota. Previous studies have examined the role of LAB and their inhibition of various enteric and foodborne pathogens (Li et al., 2017; Reuben et al., 2020). In our experiments, un-neutralized CFS of LAB showed significant antagonism against applied enteric pathogens. This could be due to the activities of organic acids they produce (Choi et al., 2018). Various effects of nCFS on inhibition of pathogens suggesting the production of bacteriocins by LAB could play a crucial role in the abolition of potentially harmful gut microbes. The LAB strains also contribute to the quality improvement of fermented foods, through deterioration and control of pathogenic bacteria, thus extending the shelf life and improving sensory quality (Beganović et al., 2014).

Fermented palm nectar contains various phytochemicals and microorganisms, including LAB, which exhibit antioxidant activity (Ghosh et al., 2015). The DPPH assay should be considered as an easy and cost-effective spectrophotometric method to evaluate the antioxidant activity of natural compounds and fermented food products. In the present study, LAB isolates showed the dose-dependent scavenging potential of DPPH as the percentage of scavenging activity increased linearly in all samples with increased concentration of DPPH and the reported IC_50_ values suggested a defensive role of TPN against oxidative stress with favorable radical quenching activities. Strains OUBN1 and OUBN5 showed higher antioxidant activity compared to previous studies by Lin et al. (2018), who reported antioxidant activity of *L. plantarum* AR501 and *P. pentosaceus* AR243.

HT-29 cell lines were used to assess the adhesion ability and anti-cancer activity of LAB isolates. *L. plantarum* group OUBN1 and *P. pentosaceous* OUBN5 showed excellent adhesive properties. MTT assay is the most widely used technique for examining new components in a short period based on their level of toxicity to cancer cells (Vuotto et al., 2014). Srinivas et al. (2017) evaluated the cytotoxic effect of yeast isolates OBS1 and OBS2 using cancer cell lines MCF7 (breast cancer) and IMR32 (neuroblastoma). Apoptosis was observed by fluorescent microscopy, which is considered a primary strategy during chemotherapy of cancer. DAPI staining method was used to observe visual symptoms of apoptosis in treated cells. Viable cells were identified as intact blue cells, while apoptotic cells were characterized by morphological changes, such as blue cells contracted with a fragmented or condensed nucleus (Haghshenas et al., 2015). HT-29 cells treated with LAB strains for 48 h showed symptoms of apoptosis, including membrane blisters, cell narrowing, nucleus fragmentation, and apoptotic body formation (Elliott et al., 2007). In the present study, CFS of all four LAB isolates showed significant cell morphological changes, including cell contraction, damage, and degeneration of cells.

## Conclusion

The present investigation involves the nutritional profiling and isolation of lactic acid bacterial strains from TPN. The nutritional profile of TPN confirmed the presence of 18 VFAs and 26 amino acids. Due to the presence of probiotic microbiota, with rich amino acid and volatile fatty acid profile, TPN the natural drink can be tried as a therapeutic agent. All the four LAB isolates viz. *L. plantarum* group OUBN1, *E. faecium* OUBN3, *P. acidilactici* OUBN4, and *P. pentosaceous* OUBN5 were found to be efficiently tolerant of low pH and bile conditions along with other severe intestinal parameters of the stomach. All four isolates showed good antimicrobial activity along with considerable antioxidant and anti-cancer activities. These strains exhibited ideal surface-binding properties, which are useful in colonizing the gastrointestinal tract and play a significant role in increasing healthy gut microbiota. The *Lactobacillus plantarum* group (reclassified as *Lactiplantibacillus plantarum*) OUBN1 and *Pediococcus pentosaceous* OUBN5 expressed potential probiotic characteristics. In conclusion, the LAB isolates from TPN established a probiotic attribute *in vitro*, thus revealing the ability to employ them as possible probiotic microbiota in food preparations.

## Data Availability Statement

The datasets presented in this study can be found in online repositories. The names of the repository/repositories and accession number(s) can be found in the article/Supplementary material.

## Author Contributions

All the experiments were carried out by NP with the help of KB. RL and BB designed the concept, monitored the work, and drafted the manuscript. All authors read and approved the final manuscript.

## Conflict of Interest

The authors declare that the research was conducted in the absence of any commercial or financial relationships that could be construed as a potential conflict of interest.
